# Smart Soup Ameliorates Cognitive Dysfunction in Diabetic Encephalopathy by Inhibiting Hippocampal Ferroptosis and Restoring Mitophagic Balance

**DOI:** 10.1155/bmri/6840529

**Published:** 2026-11-05

**Authors:** Xiaodan Yan, Dan Qin, Yan Du, Nan Li, Yu Wang, Yu Wang, Qing Yu

**Affiliations:** ^1^ School of Clinical Pharmacy Shenyang Pharmaceutical University Shenyang China syphu.edu.cn; ^2^ Research Center of Traditional Chinese Medicine Information Engineering Beijing University of Chinese Medicine Beijing China bucm.edu.cn; ^3^ The Second Affiliated Hospital of Shenyang Medical College Shenyang China; ^4^ College of Pharmacy Shenyang Pharmaceutical University Shenyang China syphu.edu.cn

**Keywords:** cognitive deficits, diabetic encephalopathy, ferroptosis, mitophagy, Smart soup

## Abstract

Diabetic encephalopathy (DE), a prevalent complication of diabetes, is strongly associated with progressive cognitive dysfunction. The iron‐dependent regulated cell death process known as ferroptosis has been implicated in the neurodegeneration observed in DE. Smart soup (SS), a classic Chinese herbal formula, is used to treat memory impairment. In this study, we evaluated the therapeutic potential of SS against DE. We found that oral administration of SS significantly ameliorated learning and memory deficits in a rat model of DE. This cognitive improvement was accompanied by the inhibition of hippocampal ferroptosis, evidenced by decreased lipid peroxidation, iron overload, and reactive oxygen species (ROS), alongside increased levels of the antiferroptotic proteins GPX4 and FTH1. Furthermore, SS treatment mitigated aberrant mitochondrial alterations and reduced the levels of mitophagy‐related proteins (PINK1 and Parkin), suggesting it alleviates the upstream damage that triggers excessive mitophagic activity. Mechanistically, SS appears to target NR3C1 (glucocorticoid receptor), downregulating its transcriptional activation of key iron metabolism factors, LCN2 and NCOA4. Collectively, our findings demonstrate that SS exerts neuroprotective effects in DE likely by suppressing ferroptosis via the NR3C1/LCN2/NCOA4 axis and improving mitochondrial homeostasis, offering a promising therapeutic strategy for this condition.

## 1. Introduction

Diabetic encephalopathy (DE) refers to a pathological complication of the central nervous system in diabetic patients, featured by neurophysiological and structural defects of the brain with different levels of cognitive impairment [1, 2]. The incidence of diabetes worldwide was estimated to be 9.3% (about 463 million people) in 2019 according to the statistic data, which, however, will rise to 10.2% (about 578 million) by 2030 and 10.9% (about 700 million) by 2045 [3]. The growing population of diabetic patients is leading to a rising prevalence of DE, which constitutes a growing threat to public health. Since its first report in 1960s, more and more attentions have been paid to DE. Unfortunately, DE still remain less understood, and currently available options for treatment are still limited [4, 5]. With these factors in mind, it is urgent to deeply study the pathogenesis of DE and develop novel therapeutics.

Traditional Chinese medicine (TCM) is a holistic system of medicine that is gaining increasing scholarly recognition within the scientific and medical communities. Many studies have reported that TCM formulas show low toxicity and have multiple biological activities, such as anti‐inflammatory and neuroprotection [6–9]. Smart soup (SS), originally recorded in “Gu Jin Yi Jian” in the Ming Dynasty of China, is such a neuroprotective TCM formulas and has been used to treat memory deficits for hundreds of years [10]. This successful application can be attributed to three traditional Chinese herbs, namely, *Radix Polygalae*, *Poriacum Radix Pini*, and *Rhizoma Acori Tatarinowii*, that have their own pharmacological efficacy against nervous dysfunctions and together make up the prescription of SS. Further, SS also shows obvious potency in the therapy of Alzheimer′s disease (AD) [11, 12]. Reduced amyloid‐*β* level and retarded neuronal loss can be observed in AD transgenic drosophila after the treatment of SS [11]. The combination of aricept with SS even exerts better therapeutic effect [12]. These studies emphasize the neuroprotective efficacy of SS against AD and provide a novel pharmaceutical approach against AD. Given that DE features AD‐like pathology, we hypothesized that SS could also improve cognitive dysfunction occurred in DE. However, the confirmed efficacy and underlying mechanism are needed to be fully investigated.

Ferroptosis, an iron‐dependent form of regulated cell death identified in 2012, is characterized by intracellular iron accumulation, glutathione (GSH) depletion, lipid peroxidation, and reduced expression of glutathione peroxidase 4 (GPX4) [13, 14]. Since its discovery, ferroptosis has been implicated in the pathogenesis of various diseases, including DE [15]. Notably, active compounds derived from Chinese herbal medicine, such as dendrobine and quercetin, have been shown to ameliorate cognitive impairment in murine models by attenuating oxidative stress, lipid peroxidation, and iron overload, thereby suppressing ferroptosis [16, 17]. These insights offer important mechanistic clues for investigating the potential relationship between SS and DE.

In this study, we employed a high‐fat diet‐induced rat model of DE and a high glucose (HG)–injured PC12 cell model to evaluate the therapeutic effects of SS on ferroptosis in DE and to delineate the underlying molecular mechanisms. Integrating network pharmacology analysis with cellular gain‐ and loss‐of‐function experiments, we identified and validated key targets of SS in the treatment of DE. These findings are anticipated to offer a safe and effective therapeutic candidate for the clinical management of DE.

## 2. Materials and Methods

### 2.1. Animals and Treatment Schedule

Eight‐week‐old male Sprague–Dawley (SD) rats were purchased and maintained in a specific pathogen‐free (SPF) animal room under the conditions as follows: temperature, 23°C–27°C; humidity, 50%–55%; and 12 h light‐dark cycle. The rats received a high‐fat diet composed of 66.5% rat maintenance diet, 20% sucrose, 10% lard, 2.5% cholesterol, and 1% sodium cholate and were free to eat and drink. The animal experiments were conducted after 1 week of acclimation. The rats in control group were received a regular diet while the other rats were received a high‐sugar and high‐fat diet for 28 consecutive days. After that, the fasting blood glucose level was detected, and the rats were considered to be diabetic when the value is larger than 7.1 mmol/L. The rats received high‐sugar and high‐fat diet were intraperitoneally injected with streptozotocin (10%, 10 *μ*L) and fasted for 12 h to induce cognitive impairment. The injection was repeated at the third day. The rats in the control group received an equal volume of sodium citrate buffer.

Then, rats in the noncontrol group with cognitive dysfunction were randomly divided into four groups with eight rats in each group, namely, DE model group, high‐dose group (DE + SS‐H), medium‐dose group (DE + SS‐M), and low‐dose group (DE + SS‐L). With the exception of the control and DE model groups, which were administered intragastrically with an equivalent volume of double‐distilled water, each treatment group was given SS by intragastric administration continuously for 4 weeks.

All animal experiments were approved by the Medicine′s Animal Ethics Committee of Shenyang Medical College. All methods were carried out in accordance with relevant guidelines and regulations, and all methods are reported in accordance with ARRIVE guidelines.

### 2.2. Preparation of SS

The SS extract was prepared by decocting a mixture of three herbs (*Rhizoma Acori Tatarinowi*, *Poriacum Radix Pini*, and *Radix Polygalae*) according to the guideline in Chinese Pharmacopoeia [18]. These herbs were purchased from Jiangyin Tianjiang Pharmaceutical Co. Ltd (Jiangyin, Jiangsu Province, China). A mixture of *Rhizoma Acori Tatarinowi* (15 g), *Poriacum Radix Pini* (15 g), and *Radix Polygalae* (15 g) was immersed in eight volumes of distilled water for 1 h, then boiled rapidly, and simmered gently for 1 h. The decoction was filtered through gauze. The residue was then decocted with six volumes of water under the same boiling and simmering conditions for another 1 h. The two filtrates were combined and concentrated to the desired volume and diluted with distilled water to target concentrations for the rats in low‐, medium‐, and high‐dose groups, respectively. Rat equivalent doses were calculated from the human clinical dose of 0.75 g/kg/day using a 6.25 conversion factor, corresponding to 1×, 2×, and 4× the human equivalent dose (4.6875, 9.375, and 18.75 g crude drug/kg/day, respectively). Therefore, the final concentrations of the low‐, medium‐, and high‐dose SS extract should be 0.059, 0.12, and 0.23 g/mL, respectively, when administered at a gavage volume of 20 mL per rat (250 g body weight). The extract was stored at 4°C until use.

### 2.3. Morris Water Maze (MWM) Test

After the last week of gavage, the MWM test was performed to evaluate the spatial learning and memory functions of rats. The MWM test was performed according to the previously reported procedures [19], and carried out in a circular pool (100 cm in diameter and 40 cm in height) filled with a depth of 50 cm opaque water. The water was colored with nontoxic black dye and kept at 25°C. The pool was divided into four quadrants. A transparent escape platform of 8 cm diameter was placed 2 cm below the water surface in a fixed quadrant of the pool. The rats were put into water from the opposite quadrant of the target quadrant and received 5 days of consecutive training. The escape latency and swimming performance were recorded. On the sixth day, the platform was removed, and the time to cross platform within 120 s was recorded. During the experiment, rat behavior was recorded by a video tracking system.

### 2.4. Network Pharmacology Analysis

The ingredients of the three herbs in SS together with their literature‐reported protein targets were collected from SymMap v2.0 (http://www.symmap.org/), in which every ingredient‐target pair is backed by PubMed‐indexed evidence. *Poriacum Radix Pini* is the sclerotium of *Poria cocos* developing around pine roots and is chemically almost identical to *Poria*, so the *Poria* record in SymMap was used. Chemical structures (SMILES), oral bioavailability, and drug‐likeness values were obtained from HERB 2.0 (http://herb.ac.cn/), and any missing information was retrieved from PubChem by compound CID. This gave 690 ingredients (208 for *Rhizoma Acori Tatarinowii*, 139 for *Radix Polygalae*, and 343 for *Poriacum Radix Pini*) connected by 10,446 curated edges to 1473, 224, and 2451 unique protein targets, respectively. Merging the three sets yielded 2486 targets.

Ferroptosis‐related genes were retrieved from FerrDb V2, restricted to human entries annotated as drivers, suppressors, or markers, yielding 471 genes. Because DE exhibits Alzheimer‐like pathology, the Open Targets AD gene set (MONDO_0004975) was used as the disease proxy, retaining 1972 genes with an overall association score equal to or more than 0.1. Since no curated DE gene set is available, robustness was assessed using five alternative gene sets: Open Targets DE (MONDO_0000489; 151 genes), Type 2 diabetes mellitus (MONDO_0005148), diabetic neuropathy (MONDO_0006626), cognitive impairment (HP_0100543), and differentially expressed genes from T2DM donor temporal‐cortex neurons (GSE161355). The KEGG ferroptosis pathway (hsa04216; 42 genes) was downloaded via the KEGG REST API. Overlaps between herb target collections and ferroptosis/disease gene sets were tested using hypergeometric tests (scipy.stats.hypergeom) against a 20,000‐gene background, with *p* < 0.05 considered significant. Core targets were defined as the intersection of the merged SS targets, ferroptosis genes, and AD genes, then grouped by ferroptosis mechanism. A herb‐target network was constructed to visualize how the three herbs share these targets.

### 2.5. Analysis of Blood Sugar Levels

The blood samples were collected from the ophthalmic venous plexus. Then, the blood was centrifuged for 15 min at 3500 rpm and 4°C to obtain serum. Serum was used for the determination of fasting blood glucose with an automatic biochemical analyzer.

### 2.6. Hematoxylin and Eosin (HE) Staining and Immunofluorescence Analysis

After MWM test, SD rats were anesthetized by intraperitoneally injected with pentobarbital sodium (40 mg/kg) (Lot Number 110320, Shanghai Experiment Reagent Co., Ltd) and euthanized. All the laboratory procedures have been applied to minimize rat sufferings, such as sterilization and fluid replenishment with normal saline (IN9000, Solarbio, Beijing, China). The rats were sacrificed via decapitation. Brain tissues of experimental rats were isolated, fixed in 4% paraformaldehyde (P1110, Solarbio, Beijing, China), embedded in paraffin, and coronally sliced into 5 *μ*m thick sections. The sections were removed from wax, rehydrated, colored, dehydrated, cleared, and stained, following the protocols of a previous study [20]. Finally, the sections were observed under an optical microscope.

Immunofluorescence staining was performed as previously described [21]. Primary antibodies included anti‐NR3C1 (1:200 dilution, ab183127, Abcam, Cambridge, United Kingdom) and anti‐NeuN antibodies (1:200 dilution, ab104224, Abcam, Cambridge, United Kingdom), while secondary antibodies included FITC‐conjugated sheep anti‐rabbit antibody (1:50, ab6717, Abcam, Cambridge, United Kingdom) and PE‐conjugated rabbit anti‐mouse antibody (1:50, ab97024, Abcam, Cambridge, United Kingdom). Antifade mounting medium with DAPI (Vector, Burlingame, California, United States) was used for nucleus staining. Images were collected using a fluorescence microscope (Olympus IX53, Japan).

### 2.7. Detection of Malondialdehyde (MDA), GSH, Superoxide Dismutase (SOD) Activity, and the Levels of Iron and Reactive Oxygen Species (ROS)

Hippocampal tissues were weighed and rinsed briefly with ice‐cold saline, then homogenized in ice‐cold saline at a ratio of 1:9 (*w*/*v*) to prepare a 10% hippocampal homogenate. The homogenate was centrifuged at 8000 rpm for 10 min at 4°C, and the supernatant was collected. Iron content was measured with a commercial iron assay kit (A039‐2‐1, Nanjing Jiancheng Bioengineering Institute, Nanjing, China) according to the manufacturer′s instructions. Sample absorbance was compared with an iron standard curve run in parallel, and results were expressed as iron concentration per milligram protein to reflect tissue iron accumulation relevant to ferroptosis. ROS levels were measured with a commercial ROS assay kit (E004‐1‐1, Nanjing Jiancheng Bioengineering Institute) based on the DCFH‐DA fluorescent probe. DCFH‐DA freely crosses cell membranes and is hydrolyzed by intracellular esterases to DCFH, which is trapped in the sample; ROS oxidizes nonfluorescent DCFH to fluorescent DCF. Fluorescence was recorded using a microplate reader at excitation/emission settings appropriate for DCF (485/525 nm), and ROS was expressed as relative fluorescence units per milligram protein. This system principally reflects H2O2, hydroxyl radical, and peroxynitrite‐derived oxidation; values are therefore reported as total ROS rather than a single species. GSH was quantified with a GSH/GSSG assay kit (S0053, Beyotime, Shanghai, China). After protein precipitation with the kit′s protein‐removal reagent, total GSH was measured by an enzymatic recycling method in which GSH reductase and NADPH regenerate GSH, and GSH reacts with DTNB to form TNB detected at 412 nm. GSSG was measured after masking GSH with the provided masking reagent, and GSH was calculated as total GSH minus 2 × GSSG. This DTNB‐based format is specific for reduced thiols under the assay conditions; strong reducing agents such as DTT or *β*‐mercaptoethanol are excluded because they interfere with the redox reaction. MDA was measured with a lipid‐peroxidation/MDA assay kit (S0131S, Beyotime) using the TBA method. Samples were mixed with TBA working solution containing an antioxidant to suppress artifactual oxidation during heating, reacted under acidic conditions, and the MDA‐TBA adduct was quantified by microplate reader (typically 532–535 nm depending on instrument) against an MDA standard curve. SOD activity was measured with a total SOD assay kit (S0101S, Beyotime) using the WST‐8 method. Superoxide was generated by the xanthine/xanthine oxidase system; WST‐8 is reduced by superoxide to a water‐soluble formazan detected at 450 nm, and SOD inhibits this reaction in proportion to its activity. One unit of SOD was defined as the amount inhibiting formazan formation by 50%, and activity was normalized to milligram protein. This format is specific for superoxide‐scavenging activity and distinguishes total SOD from other antioxidants. All measurements were performed as three independent biological replicates.

### 2.8. Preparation of the SS‐Containing Serum

Twenty SD rats were fed conventionally for 3 days and randomly divided into drug and control groups. Rats were orally administered with SS and normal saline for consecutive 3 days, respectively. One hour after the last administration, the rats were anesthetized with pentobarbital sodium. Then, blood from the abdominal aorta was taken under aseptic conditions and transferred to a 15‐mL centrifuge tube. After 2 h of standing, the blood was centrifuged at 3000 rpm for 10 min. The serum was separated, inactivated at 56°C for 30 min, and filtered using 0.22 *μ*m microporous membrane. Finally, the drug‐containing serum was stored at −20°C until use.

### 2.9. Cell Culture and Cell Viability Assay

PC12 cells are a classic and highly validated in vitro model for studying fundamental neuronal processes [22, 23], including neuroprotection, and thus used in this study. PC12 cells were purchased from the Cell Bank of the Chinese Academy of Sciences (Shanghai, China) and maintained in DMEM (Gibco, New York, United States) supplemented with 10% fetal bovine serum (Gibco, New York, United States) and antibiotics (100 U/mL penicillin and 100 *μ*g/mL streptomycin) (C0222, Beyotime, Shanghai, China) at 37°C in a 5% CO_2_ incubator. Then, cells were divided into the following groups: control group, cultured with 25 mM glucose; HG group, cultured with 100 mM glucose; low‐dose group (HG + SS‐L), cultured with 100 mM glucose and low‐dose SS (5% drug‐containing serum); medium‐dose group (HG + SS‐M), cultured with 100 mM glucose and medium‐dose SS (10% drug‐containing serum); high‐dose group (HG + SS‐H), cultured with 100 mM glucose and high‐dose SS (20% drug‐containing serum); NR3C1 overexpression group (HG + SS‐M + OE‐NR3C1), cultured with 100 mM glucose and medium‐dose SS (10%) with NR3C1 upregulated; lipocalin‐2 (LCN2) overexpression group (HG + SS‐M + OE‐LCN2), cultured with 100 mM glucose and medium‐dose SS (10%) with LCN2 upregulated; and double intervention group (HG + SS‐M + OE‐NR3C1 + sh‐LCN2), cultured with 100 mM glucose and medium‐dose SS (10%) with NR3C1 upregulated while LCN2 silenced. The treatment duration in all groups was 48 h. To perform rescue experiments, GPX4 inhibitor RSL3 (1219810‐16‐8, MCE, United States) and ROS scavenger N‐acetyl‐L‐cysteine (NAC) (616‐91‐1, MCE, United States) were added to test if inhibiting GPX4 or reducing ROS level will affect the effects of SS treatment.

Cell viability was evaluated using the Cell Counting Kit‐8 (C0038, Beyotime, Shanghai, China) according to the manufacturer′s instructions. Absorbance at 450 nm was read using a microplate reader (SpectraMax iD5, Molecular Devices, United States).

### 2.10. Western Blot

Hippocampus tissues and PC12 cells treating with SS were collected. Proteins were extracted and quantified using commercial kit (A045‐4‐2, Nanjing Jiancheng Bioengineering Institute, Nanjing, China). Then, a total of 20 *μ*g of protein was resolved by sodium dodecyl sulfate‐polyacrylamide gel (SDS‐PAGE) and transferred onto polyvinylidene difluoride (PVDF) membranes (Bio‐Rad). The membranes were immunoblotted with the primary antibodies. Primary antibodies were as follows: anti‐GPX4 (1:2000, ab125066, Abcam, Cambridge, United Kingdom), anti‐FTH1 (1:2000, ab183781, Abcam, Cambridge, United Kingdom), anti‐NR3C1 (1:2000, ab183127, Abcam, Cambridge, United Kingdom), anti‐LCN2 (1:2000, ab187370, Abcam, Cambridge, United Kingdom), anti‐NCOA4 (1:2000, ab86707, Abcam, Cambridge, United Kingdom), and anti‐GAPDH antibody (1:2000, ab263962, Abcam, Cambridge, United Kingdom). The membranes were then washed and further incubated with the goat anti‐rabbit or goat anti‐mouse secondary antibody (1:5000, ab6702 and ab6785, Abcam, Cambridge, United Kingdom). The blots were visualized using an ECL Kit (Invitrogen, United States) according to the manufacturer′s instructions. GAPDH was used as an internal control.

### 2.11. Quantitative Real‐Time PCR (qRT‐PCR)

The highly efficient RNA‐easy Isolation Reagent (R701‐01/02, Vazyme, Nanjing, China) was used to extract total RNA from PC12 cells. Concentration and purity of RNA were checked. Primers were designed and synthesized (Genscript, Nanjing, China) to amplify NR3C1, LCN2, and NCOA4 genes (Table S1). The procedures for qRT‐PCR were according to a previous description [24]. The expression level of GAPDH served as a reliable internal control.

### 2.12. Luciferase Reporter Assay

The target genes and potential binding site of the transcriptional factor NR3C1 were predicted using bioinformatics toll (LASAGNA database). To perform luciferase reporter assay, NR3C1 overexpression plasmid and LCN2 promoter reporter plasmids were constructed (Genechem Co., Ltd., Shanghai, China) and transfected into 293T cells with the reference plasmid. After 48 h, cells were collected, and the relative luciferase activities was detected using the commercial luciferase assay kit (E2920, Promega, Madison, Wisconsin, United States) according to the manufacturer′s instructions. Luminescence was then read using a CentroXS3 LB960 microplate luminometer (Berthold Technologies, Bad Wildbad, Germany).

### 2.13. Statistical Analysis

All statistical analyses were performed using the GraphPad Prism 8.0 software. Data are represented as mean ± standard deviation. Data in different groups were compared using a one‐way analysis of variance (ANOVA). Differences were considered significant at *p* < 0.05.

## 3. Results

### 3.1. Effects of SS on the Blood Glucose Levels

To determine if administration of SS bring any toxicity, body weight was monitored in rats treated with different doses of SS before the building the DE model. As shown in Figure S1, even high dose of SS did not affect the body weight of rats, indicating its nontoxicity. Then, the DE model was built on SD rats through HG and high‐fat diet feeding and streptozotocin injection. The blood glucose levels in rats in each group were monitored. As shown in Figure 1, the blood glucose level in rats in the DE model group was always significantly higher than that in the control group during the experiments (*p* < 0.05). One week post‐gavage of SS, the blood glucose levels in rats in the SS treatment groups, except the high‐dose group, showed no significant differences with that in the DE model group. Compared with the model group, the blood glucose level was significantly reduced in the rats in the SS‐treatment groups, except the low‐dose group, in 2 or 3 weeks post‐gavage of SS (*p* < 0.05). Expectedly, the blood glucose level in rats in the high‐dose SS treatment group decreased the most. These data suggest that SS could lower the glucose level in rats and administration may delay the progression of DE.

**Figure 1:**
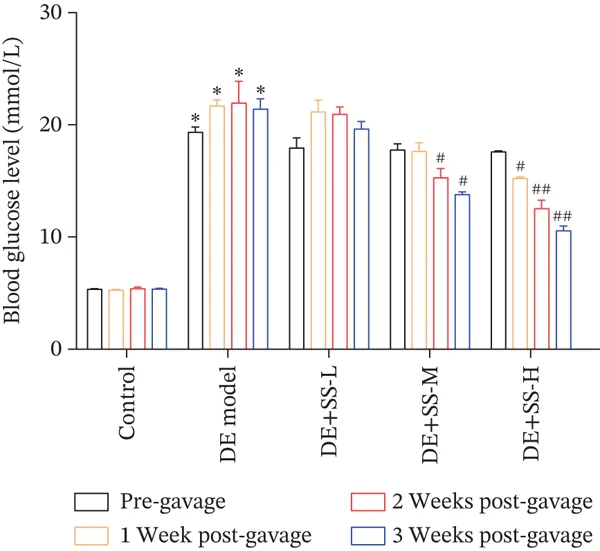
Effect of SS on the blood glucose level in SD rats with DE. Data are expressed as mean ± SD. *n* = 5 for each group. The high (18.75 g/kg), medium (9.375 g/kg), and low (4.6875 g/kg) doses of SS were expressed as SS‐H, SS‐M, and SS‐L. ∗*p* < 0.05 versus control group; #*p* < 0.05 versus DE model group; ##*p* < 0.01 versus DE model group.

### 3.2. SS Ameliorates Learning and Mnemonic Deficits of SD Rats

The MWM tests were performed to determine whether treatment with SS could enhance spatial learning and memory performance in rats. It can be seen that the time spent searching for the escape platform significantly prolonged in the DE model group compared with the control group. However, a significant reduction of the escape latency in SS‐treated rats was observed, especially in rats administrated with high‐dose SS. When the escape platform was removed, rats in the DE model group exhibited a more disordered swimming performance than that in the control groups, with a longer time spent in target quadrant fewer times crossing the platform (Figure 2A–C). By contrast, treatment with SS, especially with high dose, significantly reversed these phenomena.

**Figure 2:**
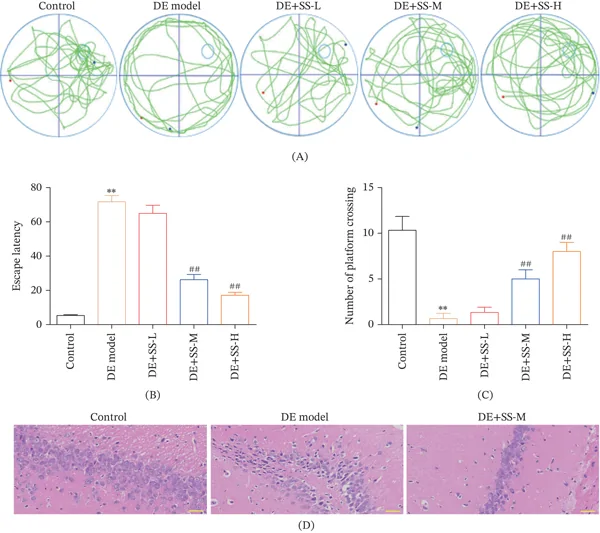
SS ameliorates mnemonic deficits of SD mice with DE. Y and Morris water maze assay were carried out to assess the mnemonic function of mice. (A) Typical swimming paths in the last training trial. *n* = 5. (B) Escape latency in the training stage. *n* = 5. (C) Platform crossing number in the testing stage. *n* = 5. Data are shown as mean ± SD. ∗∗*p* < 0.01 versus control group; ##*p* < 0.01 versus DE model group. (D) Representative hematoxylin and eosin‐stained sections of the hippocampal CA1 region. Scale bar = 50 * μ*m.

We further selected the rats in medium‐dose group for pathological analysis, and HE staining was employed to evaluate the histological changes in hippocampal CA1 region of rats. As shown in Figure 2D, more vertebral body cells and more compact arrangement of these cells were revealed in the hippocampal CA1 region of SS‐treated rats, compared with that in the DE model group and control group. These results indicated that SS intervention could improve learning and memory deficits and repair DE caused pathological damage of the hippocampus in SD rats.

### 3.3. SS Inhibits Ferroptosis in the Hippocampus of SD Rats

To figure out the effect of SS treatment on ferroptosis, the lipid peroxidation indices (GSH, SOD, and MDA), ROS level, and iron content in the hippocampus were examined. It was shown that the levels of GSH and SOD activity were significantly downregulated (*p* < 0.01), whereas the levels of MDA were significantly upregulated in the DE model group (*p* < 0.01). However, SS dose‐dependently reversed the downregulation of GSH and SOD levels and the upregulation of MDA levels (Figure 3A–C). The levels of ROS and iron were significantly higher in the DE model group (*p* < 0.01), compared with that in the control group. SS treatment mitigated the diabetes‐induced upregulation of ROS and iron levels (Figure 3D,E). These data indicate lipid peroxidation in the hippocampus of DE rats, and that the ROS and iron heavily deposited in the hippocampus of DE rats, while treatment with SS can reduce these terrible conditions.

**Figure 3:**
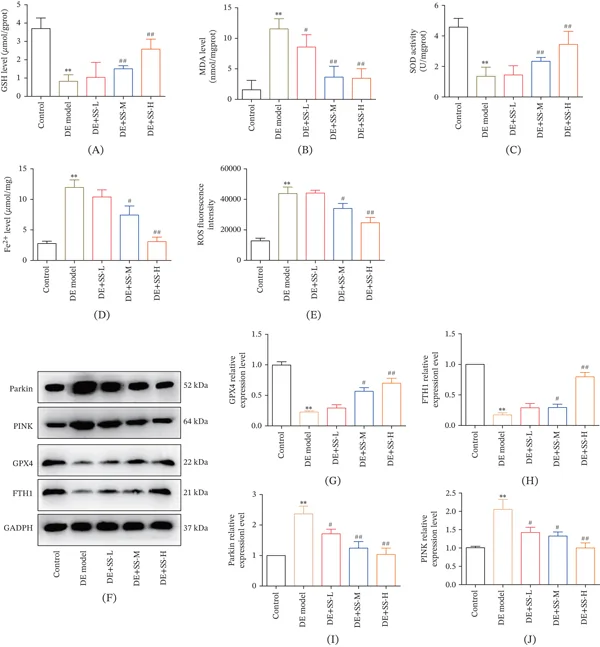
SS suppresses ferroptosis in the hippocampal neurons of SD rats with DE. The hippocampal glutathione (A) (GSH), (B) Malondialdehyde (MDA), (C) superoxide dismutase (SOD) activity, and the levels of (D) iron and (E) ROS in the hippocampus of rats in different groups (*n* = 5). (F–J) Western blotting and quantitative analysis of GPX4, FTH1, Parkin, and PINK1 in different groups. Their levels are expressed as fold change relative to the control group. Data are presented as mean ± SD. ∗∗*p* < 0.01 versus control group; #*p* < 0.05 versus DE model group; ##*p* < 0.01 versus DE model group.

GPX4 is a crucial antagonist of ferroptosis [25], and the decreased level of FTH1 could also be an inducing factor of ferroptosis [26]. In the present study, the levels of GPX4 and FTH1 in the hippocampal region of rat brains were determined. GPX4 and FTH1 expression levels were significantly lower in rats in the DE model group when compared with those in the control group (*p* < 0.01) (Figure 3F–H). SS dose‐dependently reversed the DE‐related downregulation of GPX4 and FTH1 (Figure 3F–H). These results further suggested that ferroptosis participates in the DE‐related hippocampal lesions, and SS treatment could inhibit ferroptosis through upregulating the levels of GPX4 and FTH1.

Mitochondrial dysfunction is a recognized hallmark of ferroptosis. In response to such damage, mitophagy, a selective form of autophagy that clears damaged mitochondria, can be activated as a protective mechanism to mitigate excessive ROS production. Consequently, the inhibition of ferroptosis would be expected to reduce the underlying mitochondrial injury that triggers this compensatory mitophagic response. To investigate this interplay, we assessed the expression of key mitophagy‐related proteins, PINK1 and Parkin, in the hippocampi of DE rats. Immunoblot analysis revealed that the protein levels of both PINK1 and Parkin were significantly elevated in the DE model group compared to the control group (*p* < 0.01; Figure 3F,I,J). Notably, treatment with SS attenuated this upregulation. Together, these findings suggest that SS, by inhibiting the ferroptotic cascade, may reduce the chronic pathological stimulus that drives excessive mitophagic activation, thereby contributing to the restoration of mitochondrial homeostasis in DE.

### 3.4. Network Pharmacology Analysis

The three herbs differed in target repertoire size but overlapped extensively: 185 targets were common to all three herbs, and *Rhizoma Acori Tatarinowii* and *Poriacum Radix Pini* shared 1258 targets (Figure S2). After merging, the 2486 SS targets captured 162 of the 471 ferroptosis genes and 514 of the 1972 high‐confidence Alzheimer genes (Figure 4A), representing a roughly four‐fold enrichment in ferroptosis genes (OR = 3.88, *p* = 5.9 × 10^−36^) and a nearly three‐fold enrichment in Alzheimer genes (OR = 2.87, *p* = 1.3 × 10^−68^). Each herb individually showed the same pattern (Figure 4B, all *p* < 1 × 10^−8^): *Rhizoma Acori Tatarinowii* (OR = 4.54 and 3.25 for ferroptosis and Alzheimer genes), *Radix Polygalae* (OR = 4.94 and 4.84), and *Poriacum Radix Pini* (OR = 3.91 and 2.89). Notably, *Radix Polygalae* contributed the fewest targets yet displayed the strongest enrichment.

**Figure 4:**
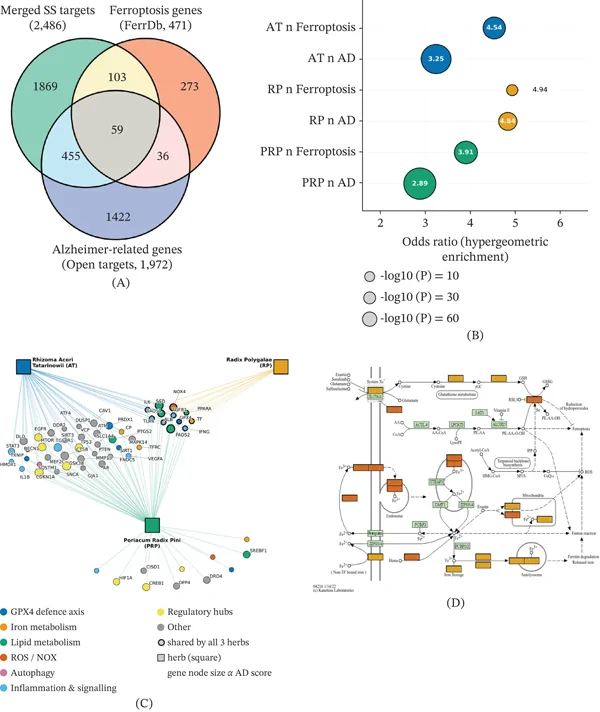
Network pharmacology analysis of SS. (A) Three‐way Venn diagram of merged SS targets (2486), ferroptosis genes (FerrDb V2, 471), and Alzheimer‐related genes (Open Targets, score ≥ 0.1, 1972); the 59 genes in the central intersection constitute the core target set. (B) Hypergeometric enrichment (odds ratio) of each herb target set in the ferroptosis and Alzheimer gene sets; bubble size is proportional to −log10(P). (C) Herb‐target bipartite network of the 59 core genes; squares represent herbs, and circles represent genes (colored by mechanistic module, sized by disease association score); bold‐rimmed nodes are genes shared by all three herbs. (D) Coverage of the KEGG ferroptosis pathway (hsa04216) by SS targets; highlighted nodes (22/42) include GPX4, HMOX1, TFRC, TF, CP, NCOA4, SLC3A2, CYBB, GCLC, and TP53.

Fifty‐nine genes lay at the intersection of SS targets, ferroptosis genes, and Alzheimer‐related genes (Figure 4A). When mapped onto the ferroptosis machinery, these genes covered the entire pathway rather than clustering at a single step (Figure 4D). The GSH‐GPX4 defense line included GPX4 itself, targeted by all three herbs along with PRDX1, PRDX6, SIRT1, SIRT3, and TXNIP. Iron handling involved transferrin (TF), transferrin receptor (TFRC), ceruloplasmin (CP), and LCN2. Lipid peroxidation substrates were represented by FADS1, FADS2, SCD, PTGS2, SREBF1, and PPARA, while the NADPH oxidases CYBB (NOX2) and NOX4 contributed enzymatic ROS sources. The list also contained autophagy adaptors (SQSTM1 and BECN1), inflammatory mediators (IL1B, IL6, IFNG, TLR4, HMGB1, and HMOX1), and upstream regulatory hubs (TP53, HIF1A, MTOR, GSK3B, and CDKN1A) (Figure S3). Consistent with this broad coverage, SS ingredients hit 22 of the 42 KEGG ferroptosis pathway components, including GPX4, HMOX1, TFRC, TF, CP, NCOA4, SLC3A2, GCLC, ATG5, ATG7, and TP53 (Figure S4). The herb–target network (Figure 4C) further showed that most of the 59 core genes were linked to two or three herbs rather than a single herb.

This enrichment was robust with respect to the selection disease gene set (Table S2). Using the DE entry (MONDO_0000489, 151 genes) instead of the Alzheimer set yielded even stronger enrichment (OR = 13.13, 95% CI 9.39–18.36, *p* = 1.2 × 10^−50^), and the T2DM and cognitive impairment sets gave consistent results (OR = 2.06 and 2.88, respectively; both *p* < 10^−32^). Under the DE set, 29 of the 59 core genes were retained, including GPX4 and LCN2 (Table S3). Only the small postmortem T2DM cortical‐neuron set (GSE161355) showed no significant enrichment, likely due to its limited sample size and cell‐type specificity rather than a lack of disease association.

Three of these core genes, namely, GPX4, LCN2, and NCOA4, guided the experiments below. GPX4, suppressed in the DE hippocampus and restored by SS (Section 3.3), is a target shared by all three herbs. LCN2, an iron‐trafficking lipocalin implicated in diabetic brain injury, belongs to the 59‐gene core set. NCOA4, the cargo receptor for ferritinophagy, is covered by SS targets in the KEGG ferroptosis pathway. In addition, NR3C1, although absent from the core set (not annotated in FerrDb V2), is listed in SymMap as a potential target of *Poriacum Radix Pini* and *Rhizoma Acori Tatarinowii* and also appears in the Alzheimer gene set. Since NR3C1 reportedly drives NCOA4 transcription and promotes ferroptosis [22, 24], we wonder whether it also acts upstream of LCN2.

### 3.5. NR3C1 Is Inhibited by SS and Transcriptionally Promotes LCN2 and NCOA4

The expression levels of NR3C1, LCN2, and NCOA4 in rat brain tissues were further investigated. Compared with the control group, the mRNA and protein levels of NR3C1, LCN2, and NCOA4 in the DE model group were significantly upregulated (*p* < 0.01) (Figure 5A–E). Similarly, SS decreased the expression levels of NR3C1, LCN2, and NCOA4 in hippocampal tissue of DE rats in a concentration‐dependent manner (Figure 5A–E). NR3C1 is a transcription factor, and its transcriptional promoting effect on NCOA4 has been well demonstrated previously [22, 24]. We then validate its potential transcriptional effects on LCN2, a 25 kDa transporter closely related to the development of DE [27]. The LASAGNA database analysis indicated that NR3C1 can indeed bind to the promoter regions of LCN2 (Figure 5F,G), as evidenced by luciferase reporter assay (Figure 5H). Notably, overexpression of NR3C1 resulted in significantly increased activity of LCN2, compared with the control (*p* < 0.01) (Figure 5H).

**Figure 5:**
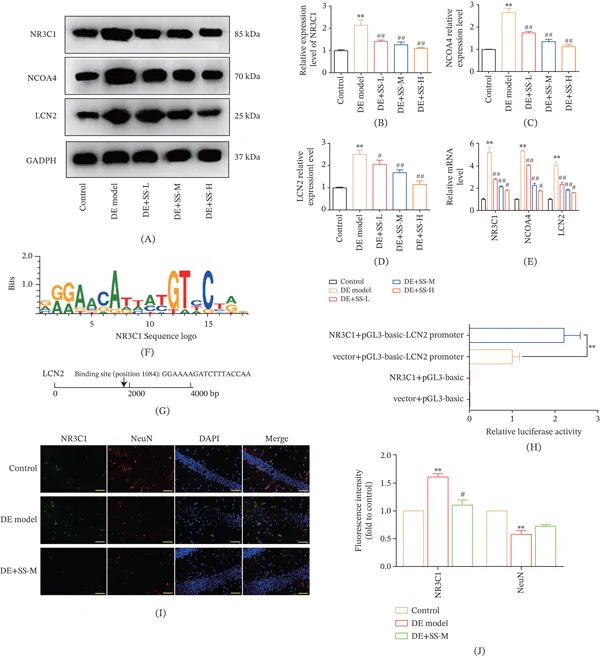
SS may target NR3C1 and regulate the expression of LCN2 and NCOA4. (A–D) Western blotting and quantitative analysis of NR3C1, LCN2, and NCOA4 in different groups. Their levels are expressed as fold change relative to the control group. Data are presented as mean ± SD. ∗∗*p* < 0.01 versus control group; #*p* < 0.05 versus DE model group; ##*p* < 0.01 versus DE model group. (E) Detection of mRNA levels of NR3C1, LCN2, and NCOA4. Data are presented as mean ± SD. ∗∗*p* < 0.01 versus control group; #*p* < 0.05 versus DE model group; ##*p* < 0.01 versus DE model group. (F) The diagram of NR3C1 binding motif (LASAGNA database). (G) The presumed NR3C1 binding site in LCN2. (H) The luciferase reporter assays revealed the direct interaction between NR3C1 and LCN2. (I) Representative images of NR3C1 and NeuN immunofluorescence staining of the hippocampal CA1 region. Scale bar = 50 * μ*m. (J) Quantitative analysis of fluorescence intensity of NR3C1 and NeuN.

The hippocampal region of rat brain tissues was also subjected to NeuN and NR3C1 double immunofluorescence staining. NR3C1 is upregulated in the hippocampus in DE rats, and the fluorescence signal of NR3C1 is overlapped with that of NeuN (Figure 5I). The fluorescence intensity of NR3C1 in the SS‐M group was markedly lower than that in the DE model group (Figure 5I,J), indicating that SS can inhibit the production of NR3C1 in hippocampal neurons. These data demonstrate that SS could regulate LCN2 and NCOA4 via directly targeting NR3C1. SS should reduce ferroptosis by inactivating NR3C1/LCN2/NCOA4 axis in DE rats.

### 3.6. SS Inhibits HG‐Induced Ferroptosis in PC12 Cells

To model diabetic neuronal injury in vitro, PC12 cells were exposed to HG (100 mM) to induce ferroptosis. HG treatment significantly decreased cell viability (*p* < 0.01), which was rescued by cotreatment with SS at all tested doses (*p* < 0.01; Figure 6A). Analysis of ferroptosis‐related markers revealed that SS suppressed the HG‐induced increase in MDA levels and reversed the HG‐induced decrease in GSH levels and SOD activity (Figure 6B–D). Furthermore, the protein levels of the antiferroptotic factors GPX4 and FTH1 were significantly downregulated by HG (*p* < 0.01) and restored by SS treatment (Figure 6E–G). Concurrently, HG exposure upregulated the mitophagy‐associated proteins PINK1 and Parkin (*p* < 0.01; Figure 6E,H,I), indicative of enhanced mitophagic flux in response to mitochondrial damage. SS treatment attenuated this upregulation. Collectively, these in vitro data demonstrate that SS inhibits HG‐induced ferroptosis and mitigates the associated mitochondrial perturbation that triggers excessive mitophagic activation.

**Figure 6:**
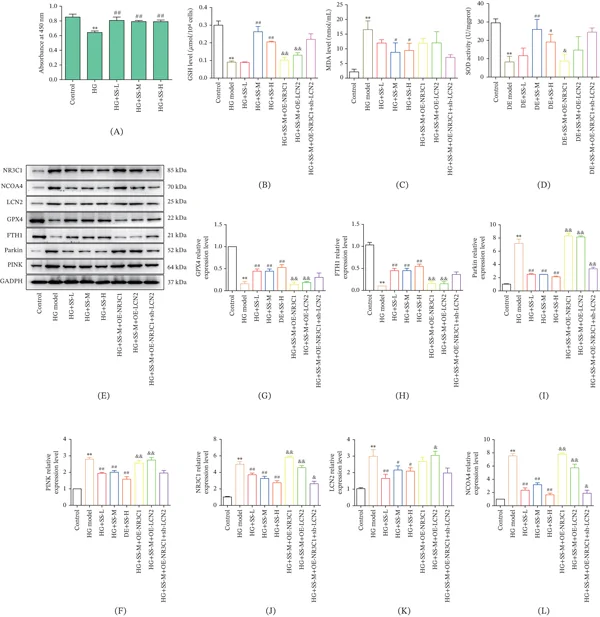
SS suppresses high glucose (HG)–induced ferroptosis and mitophagy via NR3C1/LCN2/NCOA4 axis. (A) PC12 cells were cultured with low (5% drug‐containing serum), medium (10% drug‐containing serum), and high (20% drug‐containing serum) doses of SS in the presence of 100 mM glucose for 48 h. Cell viability was examined using the CCK‐8 assay. (B) GSH and (C) MDA levels and (D) SOD activity in PC12 cells treated with SS in the presence of 100 mM glucose were detected. (E–L) Western blotting and quantitative analysis of NR3C1, NCOA4, LCN2, GPX4, FTH1, Parkin, and PINK1 levels in PC12 cells treated with SS in the presence of 100 mM glucose were detected. Their levels were expressed as fold change relative to GAPDH. All data are expressed as mean ± SD. ∗∗*p* < 0.01 versus control group; #*p* < 0.05 versus HG group; ##*p* < 0.01 versus HG group; &*p* < 0.05 versus HG + SS‐M group; &&*p* < 0.01 versus HG + SS‐M group.

The levels of NR3C1, LCN2, and NCOA4 were further analyzed to confirm the regulatory effect of SS on ferroptosis through NR3C1/LCN2/NCOA4 axis. It showed that compared with control group, the yields of NR3C1, LCN2, and NCOA4 in the HG group were significantly upregulated (*p* < 0.01) (Figure 6E,J–L). After incubated with different doses (low, medium, and high) of SS‐containing serum, the levels of NR3C1, LCN2 and NCOA4 were decreased (Figure 6E,J–L). Overexpression of NR3C1 together with SS‐containing serum treatment (medium dose, 10%) could inhibit the decreased expression of both LCN2 and NCOA4 (Figure 6J–L). Upregulation of LCN2 together with SS‐containing serum treatment could inhibit the decrease production of NCOA4 (Figure 6K,L). Overexpression of NR3C1 and suppression of LCN2 resulted in reduced level of NCOA4 (Figure 6L). Moreover, overexpression of NR3C1 and LCN2 could reduce the SS‐treatment induced effect on GSH and MDA levels and SOD activity, as well as the expression levels of GPX4, FTH1, Parkin, and PINK1 (Figure 6B–I). These results indicate that overexpression of NR3C1 reinstates the cellular state of ferroptosis and the associated mitochondrial dysfunction that drives compensatory mitophagic activation. Further, GPX4 inhibitor (RSL3) and ROS scavenger (NAC) were applied to HG‐induced PC12 cells. Both RSL3 and NAC inhibited SS′s upregulating effects on FTH1 and GPX4 (Figure 7A–D), indicating again the antiferroptotic effect of SS.

**Figure 7:**
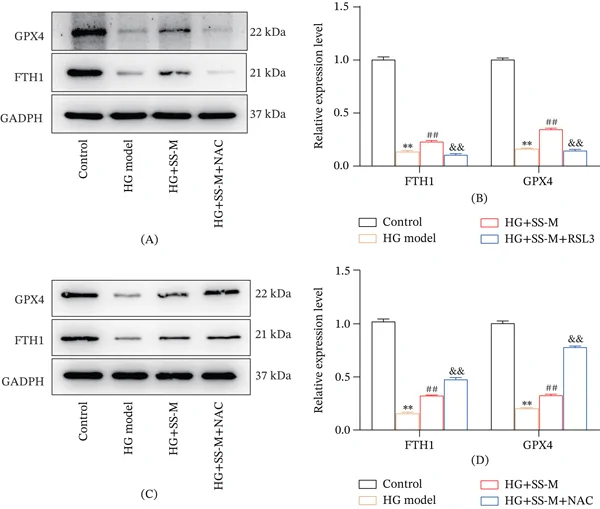
GPX4 inhibitor and ROS scavenger suppressed the therapeutic effect of SS on HG‐induced ferroptosis in PC12 cells. (A, B) Western blot and quantitative analyses of FTH1 and GPX4 levels in PC12 cells treated with SS in the presence of 100 mM glucose and GPX4 inhibitor (RSL3). (C, D) Representative Western blotting and quantitative analysis of FTH1 and GPX4 levels in PC12 cells treated with SS in the presence of 100 mM glucose and ROS scavenger (NAC). All data are expressed as mean ± SD. ∗∗*p* < 0.01 versus control group; ##*p* < 0.01 versus HG group; &&*p* < 0.01 versus HG + SS‐M group.

These results imply that SS may activate NR3C1/LCN2/NCOA4 axis, reduce mitophagy, and lead to ferroptosis inhibition and ultimate cognitive deficits improvement.

## 4. Discussion

DE is defined as the cognitive impairments and neurophysiological or structural alterations in the brain resulted from diabetes [1, 2, 28]. Clinically, DE is mainly manifested as learning and memory impairments, which may eventually lead to dementia [1, 2, 28]. These symptoms caused by DE seriously affect the quality of life of patients and create a huge burden to social medicare. With the increasing incidence of diabetes and proportion of the elderly, elucidating the molecular mechanism of DE and developing potent agents for diabetes patients are urgently needed.

SS, a Chinese translation medicine formula, is composed of three herbs officially recorded in a masterpiece published in 1576, “Gu Jin Yi Jian” [10]. It has been used in the treatment of patients with cognitive disorders for hundreds of years. In this study, we verified the therapeutic effect of SS on cognitive impairment in DE rats. The behavioral tests showed that this decoction could rescue the memory defect and improve the learning and memory capacity of DE rats. The results of histopathological staining showed that SS could improve the structure of synapses and thus enhancing the function of neurons. These data support that SS could ameliorate neurodegeneration and serve as a promising candidate to treat DE clinically.

To elucidate the neuroprotective mechanism of SS, we employed an integrated approach combining bioinformatic analysis, in vivo studies in DE model rats, and in vitro validation using the PC12 cell model. Our findings demonstrate that SS rescues cognitive dysfunction in DE by inhibiting ferroptosis. This was evidenced in vivo by decreased iron content and ROS levels, alongside increased levels of ferroptosis inhibitory factors (FTH1 and GPX4), in the brain tissue of SS‐treated DE rats. Notably, the protective role of ferroptosis inhibition aligns with reported effects of other Chinese herbal compounds [16, 17], supporting its significance as a therapeutic target in DE. In the PC12 cell model of DE, SS similarly mitigated ferroptosis markers, providing controlled mechanistic corroboration. Further investigation revealed that this antiferroptotic effect is mediated through targeting NR3C1 (the glucocorticoid receptor), which transcriptionally regulates key iron metabolism factors, LCN2 and NCOA4. Given the central role of mitochondria in ferroptosis execution, we also examined mitophagic activity. The downregulation of mitophagy‐associated proteins (PINK1 and Parkin) following SS treatment suggests that SS ameliorates the excessive activation of mitophagy. It should be pointed out that SS does not directly inhibit the core mitophagic machinery. Instead, by attenuating upstream ferroptotic injury (via the NR3C1/LCN2/NCOA4 axis) and the associated mitochondrial damage, SS reduces the pathological stimulus that triggers compensatory but ultimately detrimental mitophagic flux, thereby helping to restore mitophagic balance. In summary, our results indicate that SS effectively rescues cognitive deficits in DE. This neuroprotection is likely achieved by inhibiting ferroptosis via the NR3C1/LCN2/NCOA4 signaling axis, which in turn mitigates secondary mitochondrial damage and normalizes associated cellular clearance pathways.

We acknowledge that the present study did not include UPLC‐MS or HPLC‐based phytochemical profiling to identify the bioactive compounds in SS. This is a limitation of our work, as it leaves the relative contributions of individual compounds versus synergistic interactions to the observed therapeutic effects unclear. However, it is important to note that the network analysis adds a system‐level argument to the experimental findings of this study. Enrichment was not restricted to the formula as a whole: Each of the three herbs, analyzed on its own, was significantly overrepresented in ferroptosis genes, and the 59 core genes they share with Alzheimer‐related genes span the entire ferroptotic process, including iron import and storage, membrane lipid remodelling, ROS production, and the GPX4 defense line. This breadth fits the behavior of a multicomponent decoction, and it is consistent with what we observed in treated rats: GPX4 and FTH1 recovered, iron and ROS accumulation subsided, and the NR3C1/LCN2/NCOA4 axis was turned down. The fact that LCN2 appears in the core gene set, and that NCOA4 and the ferritinophagy machinery are covered within the KEGG ferroptosis pathway, gives network‐level support to the axis we validated experimentally. Previously published work on the constituent herbs points the same way. An *Acorus tatarinowii* extract was recently reported to suppress neuronal ferroptosis in APP/PS1 mice and A*β*‐challenged HT22 cells through Nrf2/GPX4 signaling, with reduced TFR1 and increased FPN1 expression [29]. From *Polygala tenuifolia*, tenuifolin protected HT22 cells and mice injured by D‐galactose and A*β* by restoring GPX4 and SLC7A11 while restraining ACSL4 [30] and counteracted corticosterone‐evoked ferroptosis through the same Nrf2/SLC7A11/GPX4 route [31]. Senegenin showed a comparable effect against A*β*25?35‐induced ferroptosis in PC12 cells [32]. Poria triterpenes and polysaccharides modulate ferroptosis through AMPK, PPAR*α*, and Nrf2/GPX4 in peripheral models, although neuronal systems have yet to be tested [33]. Perhaps most tellingly, Kai‐Xin‐San, which shares *Radix Polygalae*, *Rhizoma Acori Tatarinowii*, and *Poria* with SS, has been shown to inhibit ferroptosis in Alzheimer models through SIRT1/FSP1 and Keap1/Nrf2/GPX4 signaling and to normalize hippocampal TFRC, SLC7A11, GPX4, ACSL4, and FTH1 in sleep‐deprived mice [34–36]. The molecules implicated in those studies (SIRT1, GPX4, HMOX1, and TFRC) all appear among our core genes.

It is also important to note that this study utilized PC12 cells, a rat pheochromocytoma cell line, as the in vitro model. Although PC12 cells are a well‐validated system for studying neuronal survival and were therefore appropriate for our initial mechanistic investigation [22, 23], they are not of hippocampal origin. Future studies employing primary hippocampal neuronal cultures or hippocampal‐derived cell lines, such as HT‐22, will be more valuable to confirm the neuroprotection of SS against DE.

As we demonstrated in the Results section, SS treatment significantly lowers blood glucose levels in rats compared to the DE model. This phenomenon may raise the question that whether the cognitive improvement is a secondary effect of systemic glucose lowering or a direct neuroprotective effect of SS on the hippocampus. It is true that systemic glucose lowering really will contribute to cognitive improvement. However, our in vitro experiments also demonstrate that SS directly inhibits ferroptosis and enhances neuronal viability in hippocampal cultures under constant HG conditions, independent of any systemic metabolic change. This provides direct evidence for an intrinsic neuroprotective property of SS. Therefore, the cognitive benefits of SS may result from both a beneficial systemic effect (amelioration of the hyperglycemic milieu) and a crucial direct neuroprotective effect on hippocampal neurons, potentially via the inhibition of ferroptosis as delineated in this study. Future studies with longitudinal glycemic monitoring and individual‐level correlation analyses within treatment groups will help to further delineate the relative contribution of each mechanism.

The downregulation of NR3C1 is a common feature in classic stress models [37]. However, the expression level of NR3C1 was upregulated in our DE model. This phenomenon may represent a distinct, pathological response to the specific metabolic injury of DE. In this setting, heightened NR3C1 expression appears to actively drive a ferroptotic cell death program, as evidenced by its transcriptional promotion of the key ferroptosis mediator LCN2. This aligns with emerging evidence that NR3C1 activation can promote ferroptosis in other pathological contexts [24].

Although the present study demonstrated the putative role of SS in reducing cognitive impairment of SD rats by inhibiting ferroptosis in the hippocampal neurons, it has some limitations. The regulating mechanism involved in this study must be deeply investigated in the future, such as how does SS reduce the expression level of NR3C1. Although this study suggested that SS may exhibit neuroprotective activity via the NR3C1/LCN2/NCOA4 axis, whether any other pathways participate in SS‐related antiferroptotic effect remain unclear. Further studies are needed to explore other molecular pathways to fully understand SS′s antiferroptotic effect. Furthermore, the therapeutic effects and molecular mechanism of SS against some other disease are worth evaluated.

## Author Contributions

Conceptualization: Qing Yu and Yu Wang. Supervision: Qing Yu. Formal analysis: Xiaodan Yan, Dan Qin, Yan Du, Nan Li, Yu Wang, Yu Wang, and Qing Yu. Investigation: Xiaodan Yan, Dan Qin, Yan Du, Nan Li, Yu Wang, Yu Wang, and Qing Yu. Writing—original draft: Xiaodan Yan and Qing Yu. Writing—review and editing: Qing Yu. Project administration: Qing Yu.

## Funding

The study was funded by Basic Scientific Research Project of Liaoning Provincial Department of Education (Grant No LJ212610164009), Liaoning Provincial Natural Science Foundation (Grant Nos. 2024‐MSLH‐450 and 2024‐MSLH‐460), and Doctoral Startup Program of Liaoning Provincial Department of Science and Technology (Grant No. 2026‐BS‐0646).

## Conflicts of Interest

The authors declare no conflicts of interest.

## Supporting information


**Supporting Information** Additional supporting information can be found online in the Supporting Information section. Figure S1 The body weight of SD rats after treated with different doses of SS. Figure S2. The enrichment of candidate target gene pathways was analyzed in ETCM online database. Figure S3. Related diseases of target genes of three component in SS. Figure S4. The interaction among target genes of three SS ingredients. Table S1. List of primers used for qPCR analysis. Table S2. Enrichment of Smart soup (SS) merged targets (*n* = 2486) across alternative disease gene sets. Table S3. Three‐way core genes (SS targets ∩ FerrDb V2 ∩ disease gene set) retained when the diabetic encephalopathy set (MONDO_0000489) replaces the Alzheimer set.

## Data Availability

All data supporting the findings of this study are available either within the manuscript or by request.

## References

[1] McCrimmon R. J. , Ryan C. M. , and Frier B. M. , Diabetes and Cognitive Dysfunction, Lancet. (2012) 379, no. 9833, 2291–2299, 10.1016/S0140-6736(12)60360-2. 22683129

[2] Liu Y. , Li M. , Zhang Z. , Ye Y. , and Zhou J. , Role of Microglia-Neuron Interactions in Diabetic Encephalopathy, Ageing Research Reviews. (2018) 42, 28–39, 10.1016/j.arr.2017.12.005, 29247713. 29247713

[3] Saeedi P. , Petersohn I. , Salpea P. , Malanda B. , Karuranga S. , Unwin N. , Colagiuri S. , Guariguata L. , Motala A. A. , Ogurtsova K. , Shaw J. E. , Bright D. , Williams R. , and IDF Diabetes Atlas Committee , Global and Regional Diabetes Prevalence Estimates for 2019 and Projections for 2030 and 2045: Results From the International Diabetes Federation Diabetes Atlas, 9th edition, Diabetes Research and Clinical Practice. (2019) 157, 9th edition, 107843, 10.1016/j.diabres.2019.107843, 31518657. 31518657

[4] Cheng L. Z. , Li W. , Chen Y. X. , Lin Y. J. , and Miao Y. , Autophagy and Diabetic Encephalopathy: Mechanistic Insights and Potential Therapeutic Implications, Aging and Disease. (2022) 13, no. 2, 447–457, 10.14336/AD.2021.0823, 35371595. PMC8947837 35371595

[5] Xiong J. , Hu H. , Guo R. , Wang H. , and Jiang H. , Mesenchymal Stem Cell Exosomes as a New Strategy for the Treatment of Diabetes Complications, Frontiers in Endocrinology. (2021) 12, 646233, 10.3389/fendo.2021.646233, 33995278. PMC8117220 33995278

[6] Arora A. , Behl T. , Sehgal A. , Singh S. , Sharma N. , Abdellatif A. A. H. , Dailah H. G. , Bhatia S. , al-Harrasi A. , Aleya L. , and Bungau S. , Elucidating the Promising Role of Traditional Chinese Medicine in Neuroprotection Against Oxidative Stress Encompassing Alzheimer′s Disease, Environmental Science and Pollution Research. (2023) 30, no. 14, 39546–39557, 10.1007/s11356-023-25800-4, 36790717. 36790717

[7] Li W. , Yu L. , Li W. , Ge G. , Ma Y. , Xiao L. , Qiao Y. , Huang W. , Huang W. , Wei M. , Wang Z. , Bai J. , and Geng D. , Prevention and Treatment of Inflammatory Arthritis With Traditional Chinese Medicine: Underlying Mechanisms Based on Cell and Molecular Targets, Ageing Research reviews. (2023) 89, 101981, 10.1016/j.arr.2023.101981, 37302756. 37302756

[8] Tang J. L. , Liu B. Y. , and Ma K. W. , Traditional Chinese Medicine, Lancet. (2008) 372, no. 9654, 1938–1940, 10.1016/S0140-6736(08)61354-9. 18930523

[9] Leong F. , Hua X. , Wang M. , Chen T. , Song Y. , Tu P. , and Chen X. J. , Quality Standard of Traditional Chinese Medicines: Comparison Between European Pharmacopoeia and Chinese Pharmacopoeia and Recent Advances, Chinese Medicine. (2020) 15, no. 1, 10.1186/s13020-020-00357-3, 32742301. PMC7388521 32742301

[10] Ling Y. , Li Z. , Chen M. , Sun Z. , Fan M. , and Huang C. , Analysis of Multiple Constituents in Cong-Ming-Tang, a Chinese Herbal Formula for the Treatment of Amnesia, by High-Performance Liquid Chromatography With Quadrupole Time-of-Flight Mass Spectrometry, Phytochemical Analysis. (2013) 24, no. 6, 677–688, 10.1002/pca.2454, 23839964. 23839964

[11] Hou Y. , Wang Y. , Zhao J. , Li X. , Cui J. , Ding J. , Wang Y. , Zeng X. , Ling Y. , Shen X. , Chen S. , Huang C. , and Pei G. , Smart Soup, a Traditional Chinese Medicine Formula, Ameliorates Amyloid Pathology and Related Cognitive Deficits, PLoS One. (2014) 9, no. 11, e111215, 10.1371/journal.pone.0111215, 25386946. PMC4227681 25386946

[12] Wang Y. , Wang Y. , Sui Y. , Yu H. , Shen X. , Chen S. , Pei G. , Zhao J. , and Ding J. , The Combination of Aricept With a Traditional Chinese Medicine Formula, Smart Soup, May Be a Novel Way to Treat Alzheimer′s Disease, Journal of Alzheimer’s Disease. (2015) 45, no. 4, 1185–1195, 10.3233/JAD-143183, 25690664. 25690664

[13] Weiland A. , Wang Y. , Wu W. , Lan X. , Han X. , Li Q. , and Wang J. , Ferroptosis and Its Role in Diverse Brain Diseases, Molecular Neurobiology. (2019) 56, no. 7, 4880–4893, 10.1007/s12035-018-1403-3. PMC6506411 30406908

[14] Jiang X. , Stockwell B. R. , and Conrad M. , Ferroptosis: Mechanisms, Biology and Role in Disease, Nature Reviews Molecular Cell Biology. (2021) 22, no. 4, 266–282, 10.1038/s41580-020-00324-8, 33495651. PMC8142022 33495651

[15] Dixon S. J. , Lemberg K. M. , Lamprecht M. R. , Skouta R. , Zaitsev E. M. , Gleason C. E. , Patel D. N. , Bauer A. J. , Cantley A. M. , Yang W. S. , Morrison B. , and Stockwell B. R. , Ferroptosis: An Iron-Dependent Form of Nonapoptotic Cell Death, Cell. (2012) 149, no. 5, 1060–1072, 10.1016/j.cell.2012.03.042, 22632970. PMC3367386 22632970

[16] Shi Y. S. , Chen J. C. , Lin L. , Cheng Y. Z. , Zhao Y. , Zhang Y. , and Pan X. D. , Dendrobine Rescues Cognitive Dysfunction in Diabetic Encephalopathy by Inhibiting Ferroptosis via Activating Nrf 2/GPX4 Axis, Phytomedicine. (2023) 119, 154993, 10.1016/j.phymed.2023.154993, 37567006. 37567006

[17] Cheng X. , Huang J. , Li H. , Zhao D. , Liu Z. , Zhu L. , Zhang Z. , and Peng W. , Quercetin: A Promising Therapy for Diabetic Encephalopathy Through Inhibition of Hippocampal Ferroptosis, Phytomedicine. (2024) 126, 154887, 10.1016/j.phymed.2023.154887, 38377720. 38377720

[18] Yu X. , Xia K. , Wu S. , Wang Q. , Cheng W. , Ji C. , Yang W. , Kang C. , Yuan Z. , and Li Y. , Simultaneous Determination and Pharmacokinetic Study of Six Components in Beagle Dog Plasma by UPLC-MS/MS After Oral Administration of Astragalus membranaceus Aqueous Extract, Biomedical Chromatography. (2022) 36, no. 12, e5488, 10.1002/bmc.5488, 36001467. 36001467

[19] D′Hooge R. and De Deyn P. P. , Applications of the Morris Water Maze in the Study of Learning and Memory, Brain Research Reviews. (2001) 36, no. 1, 60–90, 10.1016/S0165-0173(01)00067-4, 11516773. 11516773

[20] Feldman A. T. and Wolfe D. , Tissue Processing and Hematoxylin and Eosin Staining, Methods in Molecular Biology. (2014) 1180, 31–43, 10.1007/978-1-4939-1050-2_3. 25015141

[21] Donaldson J. G. , Immunofluorescence Staining, Current Protocols in Cell Biology. (2015) 69, no. 1, 431–437, 10.1002/0471143030.cb0403s69. 26621373

[22] Oprea D. , Sanz C. G. , Barsan M. M. , and Enache T. A. , PC-12 Cell Line as a Neuronal Cell Model for Biosensing Applications, Biosensors. (2022) 12, no. 7, 10.3390/bios12070500, 35884303. PMC9313070 35884303

[23] Wu J. , Guo J. J. , Tong F. C. , Zhang S. L. , Zhang J. L. , Chen M. Y. , and Yu H. C. , Chlorogenic Acid Counteracts Mycophenolate Mofetil-Induced Neurotoxicity in Zebrafish and PC12 Cells: A Potential Bioremediation Strategy for Aquatic Pharmaceuticals, Environment International. (2025) 203, 109771, 10.1016/j.envint.2025.109771, 40946431. 40946431

[24] Zhang G. , Hu J. , Li A. , Zhang H. , Guo Z. , Li X. , You Z. , Wang Y. , and Jing Z. , Ginsenoside Rg5 Inhibits Glioblastoma by Activating Ferroptosis via NR3C1/HSPB1/NCOA4, Phytomedicine. (2024) 129, 155631, 10.1016/j.phymed.2024.155631, 38640858. 38640858

[25] Liu Y. , Wan Y. , Jiang Y. , Zhang L. , and Cheng W. , GPX4: The Hub of Lipid Oxidation, Ferroptosis, Disease and Treatment, Biochimica et Biophysica Acta (BBA)-Reviews on Cancer. (2023) 1878, no. 3, 188890, 10.1016/j.bbcan.2023.188890, 37001616. 37001616

[26] Miao R. , Fang X. , Zhang Y. , Wei J. , Zhang Y. , and Tian J. , Iron Metabolism and Ferroptosis in Type 2 Diabetes Mellitus and Complications: Mechanisms and Therapeutic Opportunities, Cell Death & Disease. (2023) 14, no. 3, 10.1038/s41419-023-05708-0, 36882414. PMC9992652 36882414

[27] Zhang W. , Chen S. , and Zhuang X. , Research Progress on Lipocalin-2 in Diabetic Encephalopathy, Neuroscience. (2023) 515, 74–82, 10.1016/j.neuroscience.2023.02.011, 36805002. 36805002

[28] Chen R. , Shi J. , Yin Q. , Li X. , Sheng Y. , Han J. , Zhuang P. , and Zhang Y. , Morphological and Pathological Characteristics of Brain in Diabetic Encephalopathy, Journal of Alzheimer’s Disease. (2018) 65, no. 1, 15–28, 10.3233/JAD-180314, 30040723. 30040723

[29] Xiong R. , Liu H. , Zhang S. , Wang L. , Zhang P. , Wang Y. , Ou X. , Wu A. , and Lai X. , Acorus tatarinowii Schott Attenuates Alzheimer′s Disease via Neuronal Ferroptosis Inhibition: A Synergistic Network Pharmacology and Multi-Omics Profiling Study, Journal of Ethnopharmacology. (2026) 356, 120829, 10.1016/j.jep.2025.120829, 41167569. 41167569

[30] Li C. , Gao F. , Qu Y. , Zhao P. , Wang X. , and Zhu G. , Tenuifolin in the Prevention of Alzheimer′s Disease-Like Phenotypes: Investigation of the Mechanisms From the Perspectives of Calpain System, Ferroptosis, and Apoptosis, Phytotherapy Research. (2023) 37, no. 10, 4621–4638, 10.1002/ptr.7930, 37364988. 37364988

[31] Xue C. , He Z. , Zeng M. , Wang Z. , Chen Q. , Qin F. , Chen M. , Ye H. , and Chen J. , The Protective Effects of Polygala tenuifolia and Tenuifolin on Corticosterone-Evoked Ferroptosis, Oxidative Stress, and Neuroinflammation: Insights From Molecular Dynamics Simulations and In Vitro Experiments, Foods. (2024) 13, no. 21, 10.3390/foods13213358, 39517142. PMC11545101 39517142

[32] Zhang H. , Zhou W. , Li J. , Qiu Z. , Wang X. , Xu H. , Wang H. , Lu D. , and Qi R. , Senegenin Rescues PC12 Cells With Oxidative Damage Through Inhibition of Ferroptosis, Molecular Neurobiology. (2022) 59, no. 11, 6983–6992, 10.1007/s12035-022-03014-y, 36068400. 36068400

[33] Liu D. , Ding J. , Li Z. , and Lu Y. , Pachymic Acid (PA) Inhibits Ferroptosis of Cardiomyocytes via Activation of the AMPK in Mice With Ischemia/Reperfusion-Induced Myocardial Injury, Cell Biology International. (2024) 48, no. 1, 46–59, 10.1002/cbin.12090, 37750505. 37750505

[34] Yan C. , Yang S. , Shao S. , Zu R. , Lu H. , Chen Y. , Zhou Y. , Ying X. , Xiang S. , Zhang P. , Li Z. , Yuan Y. , Zhang Z. , Wang P. , Xie Z. , Wang W. , Ma H. , and Sun Y. , Exploring the Anti-Ferroptosis Mechanism of Kai-Xin-San Against Alzheimer′s Disease Through Integrating Network Pharmacology, Bioinformatics, and Experimental Validation Strategy In Vivo and In Vitro, Journal of Ethnopharmacology. (2024) 326, 117915, 10.1016/j.jep.2024.117915, 38360383. 38360383

[35] Ji R. , Zang Z. , Sun Y. , Fu X. , Lan S. , Li X. , Guo L. , Liu X. , Peng C. , Wang M. , Kuang H. , and Wang Z. , Exploring the Effect of Kaixin San on Alzheimer′s Disease by Regulating the Keap 1/Nrf 2/GPX4 Signaling Pathway to Inhibit Ferroptosis Based on Metabolomics and Gut Microbiota Analysis, Fitoterapia. (2026) 190, 107162, 10.1016/j.fitote.2026.107162, 41796868. 41796868

[36] Cao Y. , Li M. , Gu L. , Zhao X. , Zhou A. , Miao Y. , Wu Y. , Ke Z. , Hu R. , Wang Z. , and Wu X. , Chinese Traditional Formula Kaixin San Suppressed Ferroptosis of Hippocampal Neurons and Cardiomyocytes in Mice With Paradoxical Sleep Deprivation, Journal of Ethnopharmacology. (2023) 304, 116034, 10.1016/j.jep.2022.116034, 36529245. 36529245

[37] Ali S. and Dwivedi Y. , Stress-Associated Changes in MiR-20b-3p as a Potential Predictor of Underlying Psychopathology in the Depressed Brain: Mechanistic Insights From a Rat Model of Chronic Restraint Stress, Molecular Neurobiology. (2025) 63, no. 1, 10.1007/s12035-025-05309-2, 41240141. PMC12619829 41240141

