# Supplementary material for: Smart Soup Ameliorates Cognitive Dysfunction in Diabetic Encephalopathy by Inhibiting Hippocampal Ferroptosis and Restoring Mitophagic Balance
Source: Biomed Res Int. 2026 Nov 5;2026:6840529. doi: 10.1155/bmri/6840529 (PMC13740681; doi:10.1155/bmri/6840529)
Supplement: Supplementary file 1 [file BMRI-2026-6840529-s001.docx]

Supplementary information for:

**“Smart soup alleviates cognitive dysfunction of diabetic encephalopathy through inhibition of hippocampal ferroptosis and regulation of mitophagy”**

Xiaodan Yan, Dan Qin, Yan Du, Nan Li, Yu Wang, Yu Wang, Qing Yu

Figure S1. The body weight of SD rats after treated with different doses of SS. Data are expressed as mean±SD. n=6 for each group. The high (18.75 g/kg), medium (9.375 g/kg), low (4.6875 g/kg) doses of SS were expressed as SS-H, SS-M, and SS-L.

Figure S2. Herb target sets and dataset overview. (A) Venn diagram of the target sets of *Rhizoma Acori Tatarinowii* (AT), *Radix Polygalae* (RP), and *Poriacum Radix Pini* (PRP). A total of 185 targets are shared by all three herbs. (B) Numbers of ingredients, ingredients with curated targets, and unique targets for each herb. (C) Overlap of each herb target set with ferroptosis genes (FerrDb V2) and Alzheimer-related genes (Open Targets, score ≥ 0.1).

Figure S3. Mechanistic grouping of the 59 core genes. Dot plot of the 59 core genes grouped by ferroptosis mechanism; the x-axis shows the Open Targets AD association score, dot size indicates the number of targeting ingredients, and colour indicates the number of targeting herbs. Stars denote KEGG hsa04216 members.

Figure S4. Attribute matrix of the 59 core genes. Each row shows the targeting herbs (dots), the AD association score (bar), the FerrDb role (driver/suppressor/marker), and KEGG hsa04216 membership (star).

Table S1. List of primers used for qPCR analysis.

| Gene | Forward | Reverse |
| --- | --- | --- |
| NR3C1 | GCAGTGGAAGGTAGACAGCA | CTGCAGTGGCTTGCTGAATC |
| LCN2 | AAGTGGCCGACACTGACTAC | AGCCCCTTGGTTCTTCCGTA |
| NCOA4 | CTGTGCTTGGAGAGACTGGG | GCCATCAGGTGCTCAGGAAT |
| GAPDH | AGGTCGGTGTGAACGGATTT | TCCCGTTGATGACCAGCTTC |

Table S2. Enrichment of Smart soup (SS) merged targets (n = 2,486) across alternative disease gene sets.

| Disease gene set | Source | Filter applied | N genes | k (SS ∩ disease) | OR (95% CI) | P value |
| --- | --- | --- | --- | --- | --- | --- |
| Alzheimer’s disease | Open Targets, MONDO_0004975 | score ≥ 0.1 | 1,972 | 514 | 2.87 (2.57–3.21) | 1.3 × 10⁻68 |
| Diabetic encephalopathy | Open Targets, MONDO_0000489 | all text-mined genes | 151 | 97 | 13.13 (9.39–18.36) | 1.2 × 10⁻50 |
| Type 2 diabetes mellitus | Open Targets, MONDO_0005148 | score ≥ 0.1 | 2,185 | 458 | 2.06 (1.84–2.31) | 3.9 × 10⁻33 |
| Diabetic neuropathy | Open Targets, MONDO_0006626 | score ≥ 0.1 | 286 | 51 | 1.54 (1.13–2.09) | 4.9 × 10⁻03 |
| Cognitive impairment | Open Targets, HP_0100543 | score ≥ 0.1 | 3,438 | 833 | 2.88 (2.63–3.17) | 4.7 × 10⁻101 |
| T2DM cortical neuron DEGs | GEO GSE161355 (6 T2DM vs 5 controls) | P<0.05 & \|log2FC\|>0.25 | 416 | 54 | 1.05 (0.79–1.40) | 3.9 × 10⁻01 |

Note: Enrichment was assessed by hypergeometric test against a background of 20,000 protein-coding genes, identical to the primary analysis. OR, odds ratio; CI, confidence interval; DEGs, differentially expressed genes. The Alzheimer’s disease set was used in the primary analysis; all other sets were used for sensitivity testing only. GSE161355 DEGs were derived from laser-capture temporal-cortex neurons of 6 donors with self-reported type 2 diabetes versus 5 non-diabetic controls (Welch’s t-test, gene-level P < 0.05 and |log2 fold change| > 0.25).

Table S3. Three-way core genes (SS targets ∩ FerrDb V2 ∩ disease gene set) retained when the diabetic encephalopathy set (MONDO_0000489) replaces the Alzheimer set.

| Gene symbol | FerrDb V2 role | Herb(s) targeting the gene | n |
| --- | --- | --- | --- |
| CREB1 | suppressor | Poria | 1 |
| FBXW7 | driver | Poria | 1 |
| GPX4 | suppressor;unclassified | Acorus, Poria, Polygala | 3 |
| GSK3B | driver | Acorus, Poria | 2 |
| HIF1A | suppressor | Poria | 1 |
| HMGB1 | driver;unclassified | Poria | 1 |
| HMOX1 | driver;suppressor;unclassified | Acorus, Poria | 2 |
| IFNG | driver | Acorus, Poria, Polygala | 3 |
| IL1B | driver | Acorus, Poria | 2 |
| IL6 | driver;suppressor | Acorus, Poria, Polygala | 3 |
| KEAP1 | driver | Acorus, Poria | 2 |
| LCN2 | suppressor | Poria | 1 |
| MAPK8 | driver | Poria | 1 |
| MTOR | suppressor | Acorus, Poria | 2 |
| NOS2 | unclassified | Acorus, Poria, Polygala | 3 |
| PTEN | driver | Acorus, Poria | 2 |
| PTGS2 | marker | Acorus, Poria | 2 |
| RB1 | suppressor | Poria | 1 |
| SIRT1 | driver;suppressor | Acorus, Poria | 2 |
| SIRT3 | driver;suppressor | Acorus, Poria | 2 |
| SIRT6 | suppressor | Poria | 1 |
| SLC2A1 | unclassified | Acorus, Poria | 2 |
| SOX2 | suppressor | Acorus, Poria | 2 |
| STAT3 | suppressor | Acorus, Poria | 2 |
| TLR4 | driver | Acorus, Poria, Polygala | 3 |
| TP53 | driver;suppressor | Acorus, Poria | 2 |
| TXNIP | unclassified | Acorus, Poria | 2 |
| VEGFA | unclassified | Acorus, Poria | 2 |
| XBP1 | unclassified | Acorus, Poria | 2 |

Note: 29 of the 59 core genes identified with the Alzheimer set are retained under the diabetic encephalopathy set, including GPX4 and LCN2. FerrDb roles: driver, ferroptosis-promoting; suppressor, ferroptosis-inhibiting; marker, ferroptosis marker; unclassified, evidence not yet classified. Herb abbreviations: Acorus, Acori Tatarinowii Rhizoma (Shichangpu); Polygala, Polygalae Radix (Yuanzhi); Poria, Poria cum Radix Pini (Fushen). Herb–target annotations are from SymMap v2.0.
